# Extended Spectrum β-Lactamases among Gram-negative bacteria of nosocomial origin from an Intensive Care Unit of a tertiary health facility in Tanzania

**DOI:** 10.1186/1471-2334-5-86

**Published:** 2005-10-15

**Authors:** Faustine Ndugulile, Roland Jureen, Stig Harthug, Willy Urassa, Nina Langeland

**Affiliations:** 1 Institute of Internal Medicine, University of Bergen, N-5021, Bergen, Norway; 2 Centre for International Health, University of Bergen, N-5021 Bergen, Norway; 3 Department of Internal Medicine, Haukeland University Hospital, N-5021, Bergen, Norway; 4 Department of Infection Control, Haukeland University Hospital, N-5021 Bergen, Norway; 5 Department of Microbiology and Immunology, Muhimbili University College of Health Sciences, Dar es Salaam, Tanzania; 6 Department of Laboratory Medicine, Alexandra Hospital, Singapore

## Abstract

**Background:**

Resistance to third generation cephalosporins due to acquisition and expression of extended spectrum β-lactamase (ESBL) enzymes among Gram-negative bacteria is on the increase. Presence of ESBL producing organisms has been reported to significantly affect the course and outcome of an infection. Therefore infections due to ESBL isolates continue to pose a challenge to infection management worldwide. The aim of this study was to determine the existence and to describe phenotypic and genotypic characteristics of ESBLs in an Intensive Care Unit (ICU) setting in Tanzania.

**Methods:**

Between October 2002 and April 2003, clinical information and samples were collected from patients suspected to have nosocomial infections in an Intensive Care Unit of a tertiary hospital in Tanzania. The isolates were identified, tested for antimicrobial susceptibility and analysed for presence of ESBL genes.

**Results:**

Thirty-nine Gram-negative bacteria were isolated from clinical samples of 39 patients. These isolates included 13 *Escherichia coli*, 12 *Enterobacter spp*, 5 *Pseudomonas spp*, 4 *Proteus spp*, 2 *Klebsiella. pneumoniae*, 2 *Citrobacter freundii *and 1 *Chryseomonas luteola*. Eleven (28.2%) of these isolates were ESBL producing. The ESBL genes characterised were SHV-12, SHV-28 and CTX-M-15. The ESBL producing isolates were more resistant to gentamicin and ciprofloxacin than non-ESBL producing isolates.

**Conclusion:**

This study shows the presence of ESBL genes among Gram-negative bacteria in the ICU setting in Tanzania. There is a need to institute strict hospital infection control policy and a regular surveillance of resistance to antimicrobial agents.

## Background

Extended spectrum β-lactamase (ESBL) enzymes have been reported in a number of species in Gram-negative bacteria. The ESBL enzymes are usually plasmid mediated and are capable of hydrolysing and inactivating a wide variety of β-lactams, including third-generation cephalosporins, penicillins and aztreonam, but are susceptible to β-lactamase inhibitors such as clavulanate, sulbactam and tazobactam [1,2]. Many ESBL producers also carry other genes that confer resistance to other antimicrobial agents such as aminoglycosides and fluoroquinolones [3,4].

Extensive use of broad spectrum antibiotics, prolonged hospitalisation, indwelling devices and severe underlying diseases have been reported as factors that have led to spread of ESBL and difficulties in managing severe infections in many parts of the world [5,6].

Treatment of ESBL-producing bacterial infections can place an added constraint on already overburden health systems in developing countries. ESBL producing organisms are reported to account for a significant proportion of intensive care infections [7]. Problems of ESBLs have led to limited, as well as expensive treatment options, and have impacted negatively on clinical outcomes [8]. Nosocomial infections due to ESBL producing organisms have been known to cause high mortality [9].

Very few studies have reported on the problem of ESBLs in Africa in general and Tanzania in particular. There have been reports of CTX -M-12 ESBL in *Klebsiella pneumoniae *in Kenya [10], TEM-53, TEM-63, SHV-2, SHV-5, SHV-19, SHV-20, SHV-21 and SHV-22 in *K. pneumoniae *in South Africa [11], CTX-M 15 and SHV-12 in Tanzania [12], SHV-12 in *Salmonella enterica *serotype Babelsberg and Enteretidis from Mali orphans [13] and TEM-3 in *S. typhimurium *in Morocco [14].

The aim of this study was to determine the existence and to describe phenotypic and genotypic characteristics of ESBLs in an Intensive Care Unit (ICU) setting in Tanzania.

## Methods

### Study design

Muhimbili National Hospital is a 1000 bed tertiary health facility with an eight-bed ICU that caters for surgical, medical and trauma emergencies. During the study period October 2002 to April 2003, 206 patients were admitted to the ICU and of these 50 specimens were collected from patients with suspected nosocomial infections, according to definitions as described by the Centers for Disease Control (CDC) [15]. In particular, infections were considered to be nosocomial if symptoms and signs appeared >48 hours following admission to the ICU.

### Bacterial strains and susceptibility testing

Specimen from 50 patients with suspected nosocomial infections were collected and cultured on blood agar (Oxoid Ltd, Basingstoke, UK) and MacConkey agar (Difco/BD Diagnostics Systems, Sparks, MI, USA) except for urine samples which were plated on Cysteine Lactose Electrolytes Deficient (CLED) agar. Isolated strains were stored in tubes containing 1.5 ml Brain Heart Infusion broth with 10% v/v glycerol at -70°C until the time of analysis. The isolates were identified using biochemical tests and confirmed using the API 20E and API 20 NE identification systems (bioMerieux, Marcy-l'Etoile, France).

The susceptibility of the *Enterobacteriaceae *to antimicrobial agents was examined by an agar diffusion method using paper disks (AB Biodisk, Solna, Sweden) containing the following antibiotic concentration [14]: amoxicillin/clavulanic acid (20/10 μg), ampicillin (10 μg), ceftriaxone (30 μg), cefuroxime (30 μg), chloramphenicol (30 μg), ciprofloxacin (5 μg), doxycycline (30 μg), gentamicin (10 μg) and trimethoprim/sulfamethoxazole (1.25/23.75 μg). The susceptibility of the *non-Enterobacteriaceae *was examined using ceftriaxone, ciprofloxacin, ceftazidime (30 μg), gentamicin, imipenem (10 μg) and tobramycin (10 μg). *E. coli *ATCC 25922 and *P. aeruginosa *ATCC 27853 were used as quality control strains.

### Testing for presence of ESBL

Gram negative bacteria with reduced susceptibility to third generation cephalosporins according to the NCCLS criteria [16], that is, zones of inhibition of = 27 mm for cefotaxime and = 22 mm for ceftazidime, were tested by three ESBL Etest strips (AB Biodisk, Solna, Sweden) on Mueller Hinton agar. Minimum Inhibitory Concentrations of cefotaxime (CT), ceftazidime (TZ) and cefepime (PM) with and without clavulanic acid were used. The inoculated plates were incubated for 16–18 hours at 37°C.

ESBL results were read either as MIC values or observation of 'phantom' zones or deformation of inhibition ellipses. Reduction of MIC by = 3 two-fold dilutions in the presence of clavulanic acid was indicative of ESBL production. Deformation of ellipses or the presence of a 'phantom' zone was also indicative of ESBL production even if the MIC ratio is <8 or cannot be read. Isolates were reported as having ESBL phenotype if one or more of the three ESBL Etests were positive.

The outcome of the test was indeterminate when MICs were outside the test range of the test device. Those strains found to be ESBL producing phenotypically by Etest were examined for the presence of the TEM, SHV and CTX-M genes by Polymerase Chain Reaction (PCR).

### Molecular analysis techniques

Isolates with ESBL phenotype were examined for the presence of *bla*_TEM_, *bla*_SHV _and blaCTX-M by PCR [17,18]. The PCR products were purified using QIAquick PCR Purification Kits (Qiagen, Hilden, Germany). The templates were sequenced using the same primers as used in the PCR amplification. The cycle sequencing parameters were 25 cycles at 96°C for 10 seconds, 58°C for 5 seconds (50°C for *bla*_CTX-M_) and 60°C for 4 minutes. The products were analysed using an ABI PRISM 3700 DNA sequencer (PE Biosystems, Warington, UK). Point mutations were accepted if present in both the forward and reverse sequences.

### Analysis of chromosomal DNA by PFGE

Pulsed-field gel electrophoresis (PFGE) was performed as described previously by Gautom et al [19]. DNA of all isolates was digested using *Xba*I (New England BioLabs, Beverly, Mass., USA) at 37°C for 4 hours, according to supplier's instructions. The slices of the digested DNA were loaded into the wells of the poured gels. Electrophoresis agarose gel (Promega, Madison, USA) with a concentration of 1% was used for different organisms in 0.5 × Tris-Borate -EDTA buffer using contour -clamped homogenous electric field apparatus (CHEF-DR III; Bio-Rad, Richmond, Calif., USA). A 48.5 kb lambda PFG marker 50 ug/ml (New England BioLabs) was used as a marker. The conditions for electrophoresis were angle, 120°; gradient, 6 V/cm; temperature, 14°C; pulse times ranging from 10 to 45 s and running time was 20 hours. The fragment patterns were interpreted as described by Tenover et al [20].

### Statistical analysis

All statistics were performed by SPSS software 11.5. Contingency table analysis was done by χ^2 ^test or two-tailed Fisher's exact test for categorical variables. P-value of <0.05 was considered statistically significant.

## Results

### Bacteriology and antimicrobial susceptibility pattern

A total of 50 bacteria were isolated from 50 patients suspected of having nosocomial infections. These consisted of 30 Urinary Tract Infections, 15 wound infections, 3 blood stream infections and 2 cases of pneumonia. Out of these 50 bacterial isolates, 39 were Gram-negative bacteria, The remaining eleven isolates were *S. aureus*. These Gram-negative isolates were identified as *Escherichia coli *(N = 13*), Enterobacter cloacae *(N = 8), *Enterobacter aerogenes *(N = 3), *Pantoea agglomerans *(N = 1), *Pseudomonas aeruginosa *(N = 5), *Proteus mirabilis *(N = 3), *Proteus vulgaris *(N = 1), *Klebsiella pneumoniae *(N = 2), *Citrobacter freundii *(N = 2) and *Chryseomonas luteola *(N = 1).

Among the ESBL producing strains a significant proportion were found to be resistant to antimicrobial agents including amoxicillin/ clavulanic acid (90.9%), doxycycline (81.5%), gentamicin (72.7%) and trimethoprim/ sulfamethoxazole (90.9%), ceftriaxone (100%) and cefuroxime (100%). The lowest levels of resistance were seen for ciprofloxacin and chloramphenicol with 45.5% each (Table 1). One *E. cloacae *and one *E. coli *were resistant to all antimicrobial agents tested. ESBL producing isolates were resistant to more antimicrobial agents than non-ESBL producing isolates. The highest rates of resistance in ESBL negative isolates were seen against ampicillin (86.4%), doxycycline (77.3%) and trimethoprim/sulfamethoxazole (63.6%). *Pseudomonas spp *were fully susceptible to imipenem, ceftazidime and tobramycin. The difference in resistance levels between ESBL and non-ESBL producing isolates for ciprofloxacin (p = 0.017), ceftriaxone (p = 0.000) and gentamicin (p = 0.003) were statistically significant (Table 1).

**Table 1:** Antimicrobial susceptibility pattern of ESBL and non-ESBL producing isolates from the ICU, Dar es Salaam, Tanzania^§^.

**Antimicrobial**	**ESBL (n = 11)**	**Non-ESBL (n = 22)**	**p-value**
	% resistant	% resistant	
Amoxicillin/Clavulanic	90.9	72.8	0.451
Ampicillin	100	86.4	0.521
Ceftriaxone	100	13.6	0.000
Cefuroxime	100	72.7	0.151
Chloramphenicol	45.5	27.3	0.514
Ciprofloxacin	45.5	4.5	0.017
Doxycycline	81.8	77.3	0.880
Gentamicin	72.7	13.6	0.003
Trimethoprim/Sulfa.	90.9	63.6	0.214

*Pseudomonas spp *and *C. luteola *were excluded in this analysis^§^.

### PCR and sequencing of ESBL genes

All Gram-negative isolates except for *Pseudomonas spp *were screened for ESBL. The eleven isolates that were phenotypically positive for ESBL were tested by PCR. Six, seven and five isolates were positive for *bla*_TEM_, *bla*_SHV _and *bla*_CTX-M_respectively (Table 2). One isolate each of *E. coli*, *K. pneumoniae *and *E. cloacae *had both *bla*_TEM _and *bla*_SHV>_. One *K. pneumoniae *isolate had only *bla*_SHV_, three isolates of *E. coli *had both *bla*_TEM _and *bla*_CTX-M _and two isolates of *E. cloacae *had both *bla*_SHV _and *bla*_CTX-M_. One *E. cloacae *isolate was phenotypically ESBL positive on Etest but was negative for *bla*_TEM_, *bla*_SHV _and *bla*_CTX-M _on PCR.

**Table 2:** Characteristics of ESBL producing isolates from ICU, Dar es Salaam, Tanzania.

**Organism**	**ESBL enzyme**	**Date of Isolation**	**Specimen**
*E. cloacae*	SHV-12, CTX-M-15	13 December 2002	pus
"	SHV-12, CTX-M-15	27 October 2002	urine
"	SHV-12	30 December 2002	urine
"	-*	16 February 2002	urine
*E. aerogenes*	SHV-12	18 October 2002	urine
*E. coli*	SHV 12	22 October 2002	urine
"	CTX-M-15	28 November 2002	urine
"	CTX-M-15	05 December 2002	urine
"	CTX-M-15	30 November 2002	pus
*K. pneumoniae*	SHV-12	30 December 2002	pus
"	SHV-28	20 February 2003	urine

*Isolate no.5 (E. cloacae) was phenotypically positive on E-test but negative on PCR.

Nucleotide sequence analysis of the isolates carrying *bla*_CTX-M _revealed that all isolates carried the CTX-M-15 gene. Of the seven isolates that had *bla *_SHV, _six had SHV-12 and one had SHV-28 (Table 2). TEM-1 was identified in TEM producing isolates.

### Genotypic relationship of the ESBL isolates

Two of the 13 *E. coli *isolated had indistinguishable restriction patterns on PFGE. These were isolates from two different patients and from different specimen (urine and pus). The two patients had at one time been together in the ICU. Other isolates tested were unrelated.

## Discussion

This study demonstrates the presence of ESBL-mediated resistance in bacteria causing infections in the ICU of a tertiary hospital in Tanzania. Despite the rise in the prevalence of ESBL in some countries, there are very few reports from Africa [10-12,14]. ESBL detection is not commonly carried out in many microbiology units in developing countries, Tanzania included. This could be attributed to lack of awareness and lack of resources and facilities to conduct ESBL identification. The high rate of resistance noted among the isolates in the present study, although few in numbers, is of serious concern. Eleven of the 39 (28.2%) of the *Enterobacteriaceae *were ESBL producing.

In this study, ESBL producing isolates were significantly more resistant to ciprofloxacin (p = 0.017), ceftriaxone (p = 0.000) and gentamicin (p = 0.003) as compared to non-ESBL producing Gram-negative isolates. Other studies have reported on cross-resistance to aminoglycosides, fluoroquinolones and trimethoprim/sulfamethoxazole in ESBL producing organisms [21,22]. Mechanisms of co-resistance are not clear, but one possible mechanism is the co-transmission of ESBL and resistance to other antimicrobials within the same conjugative plasmids [23].

This study reports on the presence of CTX-M-15 producing isolates in the ICU setting in Tanzania. CTX-M-15 gene was also found among bloodstream infection isolates from children within the same hospital [12]. This gene is widespread in European and Asian countries including Northern Italy [18], United Kingdom [24] and India [25]. The other reported gene is CTX-M-12 that was identified amongst *K. pneumoniae *isolates from cerebrospinal fluid and blood in Kenya [10]. In the present study, the CTX-M-15 gene was found in two different genera of the bacterial isolates in the ICU (*E. coli *and *E. cloacae*). Two of the CTX-M-15 producing isolates were also found to possess the SHV-12 gene. The two *E. coli *with indistinguishable PGFE restriction patterns were found to be resistant to all antimicrobials tested except for chloramphenicol.

SHV-12 was found in the two strains of *E. coli *with indistinguishable PFGE restriction patterns. SHV-12 was also found among bloodstream infection isolates from children within the same hospital [12]. This gene has also been reported in several European and Asian countries including Switzerland [26] and Thailand [27].

In the present study, this gene was found across different genera of bacterial isolates. The other SHV related genes that have been reported in Africa include SHV-2, SHV-5, SHV-19, SHV-20, SHV-21 and SHV-22 that were reported in South Africa [11].

In this study, one *K. pneumoniae *isolate was found to possess SHV-28. Initial reports of this gene came from China (GenBank accession no. AF299299). To the best of our knowledge, there are no other reports of this gene in the literature. This isolate was obtained from a urine specimen from a patient with urinary tract infection. This isolate was resistant to amoxicillin/clavulanic acid, doxycycline and trimethoprim/sulfamethoxazole and cephalosporins but sensitive to chloramphenicol, ciprofloxacin and gentamicin.

Presence of similar SHV, TEM and CTX-M genes in various non-genetically related strains suggests a possibility of horizontal transfer of these genes in the ICU. On the other hand, the two *E. coli *that produced indistinguishable restriction patterns on PFGE suggests that clonal spread of the resistant bacteria is also a potential factor in the spread of resistance in the ICU. These finding of similar ESBL genes in the ICU and in paediatric wards [12] within the same hospital could mean the ESBL genes are widespread in this hospital.

## Conclusion

This study shows that there is a presence of ESBL producing isolates in the ICU of a major hospital in Tanzania. This is also the first report of presence of SHV-28 in Africa. This study also shows that the ESBL phenotype spread in this hospital is due to multiple enzymes found in different genera of bacteria. There is a need to institute a strict hospital infection control policy and a regular surveillance of resistance to antimicrobial agents.

## Competing interests

The author(s) declare that they have no competing interests.

## Authors' contributions

FN was the principal investigator, participated in the planning and execution of the study, performed data entry and data analysis, laboratory work and was the main responsible author. RJ participated in the planning of the study, contributed to the writing process and provided advice with the laboratory work. SH and WU participated in the planning of the study and contributed to the writing process. NL was the project coordinator and participated in planning, data analysis and writing.

All authors have read and approved the final manuscript.

## Pre-publication history

The pre-publication history for this paper can be accessed here:


